# Survival characteristics and prognostic importance of echocardiographic measurements of right heart size and function in dogs with pulmonary hypertension

**DOI:** 10.1111/jvim.15826

**Published:** 2020-06-05

**Authors:** Lance C. Visser, James E. Wood, Lynelle R. Johnson

**Affiliations:** ^1^ Department of Medicine and Epidemiology School of Veterinary Medicine, University of California, Davis Davis California USA

**Keywords:** canine, Doppler echocardiography, mitral valve disease, postcapillary, precapillary, pulmonary vascular resistance

## Abstract

**Background:**

The clinical relevance of echocardiographic measurements of right heart size and function in dogs with pulmonary hypertension (PH) is unknown.

**Objective:**

To determine if echocardiographic measurements of right heart size and right ventricular (RV) function are associated with survival times in dogs with PH.

**Animals:**

Eighty‐two client‐owned dogs.

**Methods:**

Retrospective study where data from medical records and baseline echocardiographic examinations were collected and measured in a standardized manner. Owners or primary veterinarians were contacted for outcome data.

**Results:**

Enlargement of the right atrium (88%), RV (69%), and pulmonary artery (72%) was common. One‐third of the cases had reduced RV function quantified by two‐dimensional echocardiography‐derived tricuspid annular plane systolic excursion (TAPSE). Decreased TAPSE was significantly (*P* = .008) more common in dogs with PH not secondary to left heart disease (LHD; 43%) compared to dogs with PH secondary to LHD (14%) but median survival times (182, 95% confidence interval [CI] = 39‐309 versus 298, 95% CI = 85‐314 days, respectively) were not significantly different (*P* = .78). Right atrial area (hazard ratio [HR] = 2.72, 95% CI = 1.58‐4.70), TAPSE < 3.23 mm/kg^0.284^ (HR = 2.19, 95% CI = 1.28‐3.74), and right heart failure (HR = 2.05, 95% CI = 1.18‐3.57) were independently associated with shorter survival time (*P* ≤ .04).

**Conclusions and Clinical Importance:**

Right atrial area, RV function (TAPSE < 3.23 mm/kg^0.284^), and right heart failure offer clinically relevant prognostic information in dogs with PH. Results support the quantitative assessment of right heart size and function in dogs with PH.

**Abbreviations**
AT/ET: acceleration time of pulmonary artery blood flow indexed to ejection time
LA: left atrium/atrial
LA/Ao: left atrium‐to‐aortic root dimension
LAD: left atrial dimension
LHD: left heart disease
LVIDd: left ventricular internal dimension at end‐diastole
MMVD: myxomatous mitral valve disease
PG: pressure gradient
PH: pulmonary hypertension
PV/Ao: pulmonary valve indexed to the aortic root
RA: right atrium/atrial
RAA: right atrial area
RPAD: right pulmonary artery distensibility
RV: right ventricle/ventricular
RVAd: right ventricular area at end‐diastole
RVIDd: right ventricular internal dimension at end‐diastole
TAPSE: tricuspid annular plane systolic excursion
TRPG: tricuspid regurgitation pressure gradient
TRV: tricuspid regurgitation velocity

## 1 INTRODUCTION

Pulmonary hypertension (PH) is a secondary complication of many commonly encountered cardiopulmonary disorders and is an increasingly recognized clinically important finding in dogs.^1^, ^2^, ^3^, ^4^, ^5^ It is commonly associated with and can lead to clinical signs such as exercise intolerance, breathing difficulty, and exertional syncope and can cause right heart failure.^4^, ^5^, ^6^, ^7^, ^8^, ^9^, ^10^ Reports have suggested that a tricuspid regurgitation pressure gradient (PG) > 55 mm Hg and ≥47 mm Hg is associated with an increased risk of death in dogs with myxomatous mitral valve disease (MMVD)^5^ and respiratory disease/hypoxia,^4^ respectively.

Veterinarians are heavily reliant on echocardiography to provide a clinical diagnosis of PH. Doppler echocardiography‐derived tricuspid regurgitation velocity (TRV) and thus tricuspid regurgitation PG calculated using the simplified Bernoulli equation (PG = 4 × velocity [m/s]^2^) is the primary metric used to estimate pulmonary arterial pressure and helps to assess the echocardiographic probability a dog has PH.^2^ Doppler echocardiography has limitations and therefore might be inaccurate^11^ and imprecise.^12^, ^13^ Several additional echocardiographic measurements of right heart size and function can be utilized to aid the echocardiographic assessment of PH. These measurements are largely based on characteristic cardiac changes that occur secondary to PH and have been previously evaluated in dogs diagnosed with PH. For example, the pulmonary artery might dilate,^7^, ^14^ exhibit reduced distensibility (quantified by the right pulmonary artery distensibility [RPAD] index),^14^, ^15^, ^16^, ^17^ or both. The blood flow profile in the pulmonary artery might exhibit a shortened acceleration time when indexed to ejection time (AT/ET).^7^, ^14^, ^18^ The right atrium (RA),^19^ right ventricle (RV),^20^ or both might become dilated. The RV might exhibit systolic dysfunction when, for example, quantified by tricuspid annular plane systolic excursion (TAPSE).^21^, ^22^ In addition to assessing the likelihood a dog has PH, these quantitative right heart measurements might help determine the clinical severity and prognosis of dog with PH.

Longitudinal studies on dogs with PH that describe survival characteristics and the prognostic value of echocardiographic measurements of right heart size and function will likely provide clinically useful information, regardless of the underlying etiology of disease. Such measurements have been demonstrated to be powerful prognostic indicators in humans with PH.^23^, ^24^ Identifying echocardiographic variables associated with survival time in dogs might aid clinical recommendations, identify future therapeutic targets, or both. Therefore, the primary objective of our study was to determine if echocardiographic measurements of right heart size and function are associated with survival times in dogs with PH. We also sought to compare echocardiographic measurements of right heart size and function in dogs with PH secondary to left heart disease (LHD) to dogs with PH not secondary to LHD. Last, we sought to determine the prevalence of right heart remodeling and RV dysfunction in dogs diagnosed with PH.

## 2 MATERIALS AND METHODS

### 2.1 Animals

The echocardiography database at the University of California, Davis, Veterinary Medical Teaching Hospital was searched for dogs diagnosed with PH between January 2015 and July 2018. Dogs were enrolled on a consecutive basis provided the inclusion criteria and none of the exclusion criteria were met.

#### 2.1.1 Inclusion criteria

Dogs had to have a complete two‐dimensional and Doppler echocardiographic examination using standardized imaging planes^25^ that included adequate visualization of right heart structures. Dogs had to have at least three tricuspid regurgitation jets recorded to allow measurement of TRV. The peak TRV determined using continuous wave Doppler that was recorded in the echocardiography report had to be >3.4 m/s.^2^ The TRV profiles had to be clearly visible and their contour had to comply with known hemodynamic principals.

#### 2.1.2 Exclusion criteria

Dogs were excluded if they had a RV outflow tract obstruction (eg, pulmonary valve stenosis), if they had intracardiac heartworms and underwent interventional heartworm retrieval, if they were diagnosed with congenital cardiac shunt, or if they had a sustained or clinically important arrhythmia (eg, dogs that underwent treatment or pacing for an arrhythmia).

### 2.2 Classification of PH

Dogs were classified with PH secondary to LHD if they had LHD (eg, MMVD) and unequivocal echocardiographic left atrial enlargement^2^ according to two different measurements of left atrial size: (1) left atrial‐to‐aortic root dimension (LA/Ao) > 1.7 and (2) left atrial dimension (LAD) normalized to bodyweight > 1.6.^26^ Because of the retrospective study design and variable diagnostic evaluations, all remaining dogs were grouped as dogs with PH not secondary to LHD.

### 2.3 Echocardiographic measurements and calculations

All echocardiographic examinations were performed by one of the authors (a board‐certified cardiologist) or a cardiology resident trained by the author. All echocardiographic assessments, measurements, and calculations were performed by a single investigator (L. C. V.) at an off‐cart workstation (Syngo Dynamic Workplace, Siemens Medical Solutions, Inc, Malvem, Pennsylvania). All echocardiographic studies were measured in the same order. The TRV recorded for study purposes was measured last. It was measured using continuous‐wave Doppler recordings from the imaging plane that permitted the fastest jet profile. The TRV was converted to a tricuspid regurgitation pressure gradient (TRPG) using the simplified Bernoulli equation: TRPG = 4 × TRV (m/s)^2^. Regurgitant jets were measured at the dense outer edge of the velocity profile and measurements of fine linear signals (feathered edge) were avoided.^27^, ^28^, ^29^ Values for each echocardiographic variable consisted of the average of 3, usually consecutive, cardiac cycles. Measurements of cardiac structures were made using two‐dimensional echocardiography.

Linear measurements of the left atrium (LA), left ventricle, and RV were performed from the right parasternal 4‐chamber long‐axis imaging planes. Maximum LAD was measured midchamber at end (ventricular)‐systole with a line extending from the region of the fossa ovale to the internal reflection of the hyperechoic pericardium in the far field approximately parallel to the mitral annulus.^26^, ^30^ Left ventricular internal dimension at end‐diastole (LVIDd) was measured at the level of the chordae tendineae perpendicular to the long/major‐axis of the ventricle.^26^, ^30^ From the same image and in the same manner, right ventricular internal dimension at end‐diastole (RVIDd) was measured.

Left atrial and aortic dimensions were measured from the right parasternal short‐axis view at the level of the aortic root.^31^, ^32^ Both linear measurements were made in early diastole, that is, the earliest frame in which the closed aortic valve cusps could be visualized. The aortic root measurement was made starting from the midpoint of the convex curvature of the internal wall of the right aortic sinus of Valsalva and continuing along the commissure of the left and noncoronary cusps to the junction of the aortic wall, left coronary cusp, and noncoronary cusp. The LA measurement was made from the internal border of the LA extending from and parallel to the commissure between left and noncoronary cusp (ie, continued along the trajectory of the aortic root measurement) to the internal border of the distant LA wall in the far field. If a pulmonary vein entered the LA at the desired measurement point, the line was placed on what was considered to be an extrapolation of the atrial border.^31^

The right parasternal short‐axis basilar view optimized for the RV outflow tract that permitted adequate visualization of the pulmonary trunk and right pulmonary artery was used for the following measurements. Diameter of the pulmonary valve was measured in early diastole and indexed to the aorta root measurement (PV/Ao) as described above for LA/Ao. Pulmonary regurgitation velocity was measured in early diastole, if present. The ratio of acceleration time to ejection time (AT/ET) of pulsed‐wave Doppler recordings of blood flow was measured at the level of the pulmonary valve.^18^ Right pulmonary artery distensibility index was measured as previously described.^14^ Internal diameter of the minimum diastolic and maximum systolic right pulmonary artery was quantified at the same location on the right pulmonary artery. The RPAD index was calculated according to the formula: RPAD index = (maximum diameter of the right pulmonary artery in systole − minimum diameter of the right pulmonary artery in diastole)/maximum diameter of the right pulmonary artery in systole.

A left apical 4‐chamber view, usually optimized for the right heart, was utilized for measurements of right atrial and ventricular size and RV function. Maximum right atrial (end‐systolic) and ventricular (end‐diastolic) area (RAA and RVAd, respectively) were quantified using planimetry as previously described.^33^ Right ventricular systolic function was quantified with TAPSE using two‐dimensional echocardiography.^34^

### 2.4 Clinical progress and survival

Study investigators contacted dog owners, primary care veterinarians, or both to determine the outcome of each dog (alive, natural death, or euthanasia) if this could not be gathered from our hospital's medical records. Survival time was counted from the day of the echocardiographic diagnosis of PH to either the day of death or, if alive, to the start of data collection (August 30, 2019). Because of the challenge of attributing death directly to PH, the end point of the study was death/euthanasia of any cause (all‐cause mortality).

### 2.5 Statistical analysis

Statistical analyses were performed using commercially available software (Prism 7, GraphPad Software, Inc, La Jolla, California and MedCalc Statistical Software, MedCalc Software bvba, Ostend, Belgium). Descriptive statistics were generated and the Shapiro‐Wilk test was used for normality testing of continuous data. If normally distributed, continuous data were reported as mean (SD) and compared with an independent *t* test. If normality testing failed, continuous data were reported as median (interquartile range) and compared with a Mann‐Whitney test. Categorical data were summarized as proportions and percentages and were compared with a Chi‐squared test.

Effects of the following baseline clinical and echocardiographic variables on survival were evaluated using Kaplan‐Meier survival curves and log‐rank tests: (1) age, (2) PH secondary to LHD versus other causes, (3) right heart failure, (4) syncope, (5) TRV, (6) RAA, (7), RVAd, (8) TAPSE, (9) PV/Ao, (10) RPAD index, and (11) AT/ET. To evaluate for collinearity of the echocardiographic variables, all were compared using Spearman rank correlation analysis. Dogs that were lost to follow up were right‐censored after their last known contact. Dogs that were alive at the end of the study period were also right‐censored. Dogs that died before hospital discharge at the time of their echocardiographic diagnosis of PH were excluded from the survival analysis.

Cutoffs for continuous variables were chosen based on clinical relevance and the cited veterinary literature as follows: TRPG > 75 mm Hg (severe PH),^1^ RAA > 0.76 cm^2^/kg^0.71^,^33^ RVAd > 1.33 cm^2^/kg^0.62^,^33^ TAPSE < 3.23 mm/kg^0.284^,^34^ PV/Ao > 1.0,^7^, ^14^ RPAD index < 0.30,^14^, ^15^ and AT/ET < 0.30.^7^, ^14^, ^18^ Univariate Cox proportional‐hazards regression was used to define predictor variables to enter into the multivariable analysis based on a *P* < .20. Two separate multivariable Cox proportional‐hazards regression analyses were performed; one using continuous echocardiographic predictor variables and one using the aforementioned clinical cutoffs (categorical echocardiographic predictor variables). Multivariable analyses were performed using a forward stepwise approach. Following the multivariable Cox proportional‐hazards regression analysis on the continuous echocardiographic predictor variables, a post hoc analysis of RAA was performed in order to explore clinically relevant cutoffs for RAA. Cutoffs were explored by dividing RAA into groups based on quartiles, and the effect on survival of these cutoffs was evaluated using Kaplan‐Meier survival analysis and Log‐rank tests. For all analyses, statistical significance was set at *P* < .05.

## 3 RESULTS

### 3.1 Baseline clinical and echocardiographic data

Eighty‐eight dogs met the inclusion criteria. However, 3 dogs were excluded because they had intracardiac heartworms and underwent interventional retrieval. Three dogs were excluded because of a congenital cardiac shunt; 1 with a left‐to‐right shunting atrial septal defect and 2 with a right‐to‐left shunting patent ductus arteriosus. Thus, 82 dogs were enrolled in our study and a summary of clinical findings is presented in Table 1. Breeds that were represented more than once included the Chihuahua (14), Shih Tzu (7), Maltese (6), Pomeranian (5), Cavalier King Charles Spaniel (5), Miniature Dachshund (3), Border Collie (3), Beagle (2), West Highland White Terrier (2), Boston Terrier (2), Pekingese (2), Yorkshire Terrier (2), Miniature Poodle (2), and Golden Retriever (2). Twelve dogs were mixed breed. Most of the dogs exhibited clinical signs (77 of 82; 94%) including syncope (40 of 82; 49%), breathing difficulty or tachypnea (35 of 82; 43%), cough (21 of 82; 26%), abdominal distension (15 of 85; 18%), lethargy (7 of 82; 9%), or exercise intolerance (7 of 82; 9%). Many dogs were being managed with a variety of medications at the time of diagnosis of PH (Table 1). Three dogs (4%) with PH not secondary to LHD were receiving sildenafil at the time of evaluation.

**TABLE 1:** Baseline clinical data from 82 dogs with pulmonary hypertension

Clinical variable	All dogs (n = 82)	PH not secondary to LHD (n = 54)	PH secondary to LHD (n = 28)
Bodyweight (kg)	6.1 (3.9‐9.2)	6.3 (4.3‐9.8)	5.8 (3.8‐9.0)
Age (y)	12.3 (10.6‐14.1)	12.2 (10.3‐14.1)	12.5 (11.0‐14.1)
Female: number (%)	42 (51%)	30 (55%)	12 (43%)
Syncope: number (%)	40 (49%)	24 (44%)	16 (57%)
Right heart failure: number (%)	23 (28%)	10 (18%)	13 (46%)
Left heart failure: number (%)	n/a	n/a	12 (43%)
Furosemide: number (%)	26 (32%)	11 (20%)	15 (54%)
Pimobendan: number (%)	21 (26%)	6 (11%)	15 (54%)
Sildenafil: number (%)	3 (4%)	3 (5%)	0 (0%)
ACE inhibitor: number (%)	23 (28%)	9 (17%)	14 (50%)
Spironolactone: number (%)	5 (6%)	0 (0%)	5 (18%)
Bronchodilator: number (%)	4 (5%)	3 (5%)	1 (3%)
Clopidogrel: number (%)	3 (4%)	3 (5%)	0 (0%)
Cough suppressant: number (%)	3 (4%)	2 (4%)	1 (3%)
Prednisone: number (%)	3 (4%)	2 (4%)	1 (3%)

Abbreviations: ACE, angiotensin converting enzyme; LHD, left heart disease; n/a, not applicable; PH, pulmonary hypertension.

Forty dogs (49%) were tested for *Dirofilaria immitis* antigen, of which, 1 tested positive, however no heartworms were visible with echocardiography. Left heart disease was documented in 28 of 82 dogs (34%) all of which were diagnosed with MMVD. The majority of dogs (54 of 82; 66%) had PH that was not associated with LHD.

A summary of the echocardiographic data for all dogs at the time of the diagnosis of PH is presented in Table 2. Right atrial, RV, and pulmonary artery enlargement were common, present in 88%, 69%, and 72%, respectively. One‐third of dogs had RV systolic dysfunction as evidenced by TAPSE < 3.23 mm/kg^0.284^. Dogs with PH not secondary to LHD had significantly (*P* < .001) lower TAPSE (3.30 [2.74‐4.48] mm/kg^0.284^) compared to dogs with PH secondary to LHD (5.38 [4.11‐6.30] mm/kg^0.284^). Also, the proportion of dogs with reduced TAPSE (<3.23 mm/kg^0.284^) was significantly (*P* = .008) higher in dogs with PH not secondary to LHD (23 of 53; 43%) compared to dogs with PH secondary to LHD (4 of 28; 14%). Decreased RPAD and AT/ET were also common, occurring in 87% and 62% of dogs, respectively. Tricuspid annular plane systolic excursion, PV/Ao, and AT/ET were all significantly (*P* ≤.006) increased in dogs with PH secondary to LHD (5.38 [4.11‐6.30] mm/kg^0.284^, 1.18 [1.08‐1.31], 0.31 [0.07], respectively) when compared to dogs with PH not secondary to LHD (3.30 [2.74‐4.48] mm/kg^0.284^, 1.07 [0.98‐1.16], 0.26 [0.08], respectively). Conversely, RVIDd/LVIDd was significantly (*P* < .001) decreased in dogs with PH secondary to LHD (0.56 [0.36‐0.71]) when compared to dogs with PH not secondary to LHD (0.93 [0.70‐1.23]).

**TABLE 2:** Baseline echocardiographic data from 82 dogs with pulmonary hypertension

Echocardiographic variables^a^	All dogs	PH not secondary to LHD	PH secondary to LHD	*P* value
TRV (m/s)	4.15 (3.78‐4.65)	4.31 (3.81‐4.75)	3.97 (3.75‐4.44)	.19
PRV (m/s)	3.0 (0.4)	3.08 (0.51)^b^	2.90 (0.17)^c^	.34
RAA (cm^2^/kg^0.71^)	1.16 (0.90‐1.50)	1.16 (0.92‐1.53)	1.13 (0.85‐1.40)	.43
RAA > 0.76: proportion (%)	71/81 (88%)	45/53 (85%)	26/28 (93%)	.30
LAD (cm/kg^0.308^)	1.39 (1.16‐1.91)	1.19 (1.09‐1.38)	2.09 (1.82‐2.37)	n/a
LA/Ao	1.47 (1.30‐2.19)	1.40 (1.21‐1.47)	2.40 (2.14‐2.76)	n/a
RVAd (cm^2^/kg^0.62^)	1.47 (0.39)	1.48 (0.40)	1.44 (0.40)	.60
RVAd > 1.33^b^: proportion (%)	56/81 (69%)	36/53 (68%)	20/28 (71%)	.74
RVIDd/LVIDd	0.75 (0.57‐1.04)	0.93 (0.70‐1.23)	0.56 (0.36‐0.71)	**<.001**
TAPSE (mm/kg^0.284^)	4.07 (3.08‐5.26)	3.30 (2.74‐4.48)	5.38 (4.11‐6.30)	**<.001**
TAPSE < 3.23: proportion (%)	27/81 (33%)	23/53 (43%)	4/28 (14%)	**.008**
PV/Ao	1.11 (1.00‐1.23)	1.07 (0.98‐1.16)	1.18 (1.08‐1.32)	**.002**
PV/Ao > 1.0: proportion (%)	59/82 (72%)	33/54 (61%)	26/28 (93%)	**.002**
RPAD index (%)	0.19 (0.09)	0.18 (0.09)	0.20 (0.08)	.30
RPAD index < 0.30: proportion (%)	68/78 (87%)	47/53 (89%)	21/25 (84%)	.56
AT/ET	0.28 (0.08)	0.26 (0.08)	0.31 (0.07)	**.006**
AT/ET < 0.30: proportion (%)	47/76 (62%)	36/50 (72%)	11/26 (42%)	**.01**

*Note:* Continuous data reported as mean (SD) if normally distributed and median (interquartile range) if non‐normality distributed. *P* values represent the comparison of PH not secondary to LHD to PH secondary to LHD. Bolded values denote statistical significance.

Abbreviations: Ao, aortic root; AT/ET, acceleration time to ejection time ratio of pulmonary artery flow; LA, left atrium; LAD, left atrial dimension; LHD, left heart disease; LVIDd, left ventricular internal dimension at end‐diastole; n/a, not applicable; PH, pulmonary hypertension; PRV, pulmonary regurgitation velocity; PV, pulmonary valve; RAA, right atrial area; RPAD, right pulmonary artery distensibility; RVAd, right ventricular area at end‐diastole; RVIDd, right ventricular internal dimension at end‐diastole; TAPSE, tricuspid annular plane systolic excursion; TRV, tricuspid regurgitation velocity.

^a^ Not all echocardiographic measurements could be performed on all dogs. Refer to the listed proportions under each measurement.

^b^ Present in 13 of 54 (24%) dogs.

^c^ Present in 8 of 28 (29%) dogs.

### 3.2 Survival analysis

Four dogs were excluded from the survival analysis because of euthanasia before hospital discharge; all had PH not secondary to LHD. Sixty‐seven (86%) dogs died during the study period, which lasted 4 years and 8 months. Forty‐four dogs (66%) were euthanized and 23 (34%) died naturally. Eleven dogs were right‐censored; 5 were still alive and 6 were lost to follow up. Overall median survival time (n = 78 dogs) was 192 days (95% confidence interval [CI] = 85‐314). There was no significant difference (*P* = .78) between median survival time for dogs with PH not secondary to LHD (182 days [95% CI 39‐309]; n = 50) compared to dogs with PH secondary to LHD (298 days [95% CI 85‐314]; n = 28).

**FIGURE 1:**
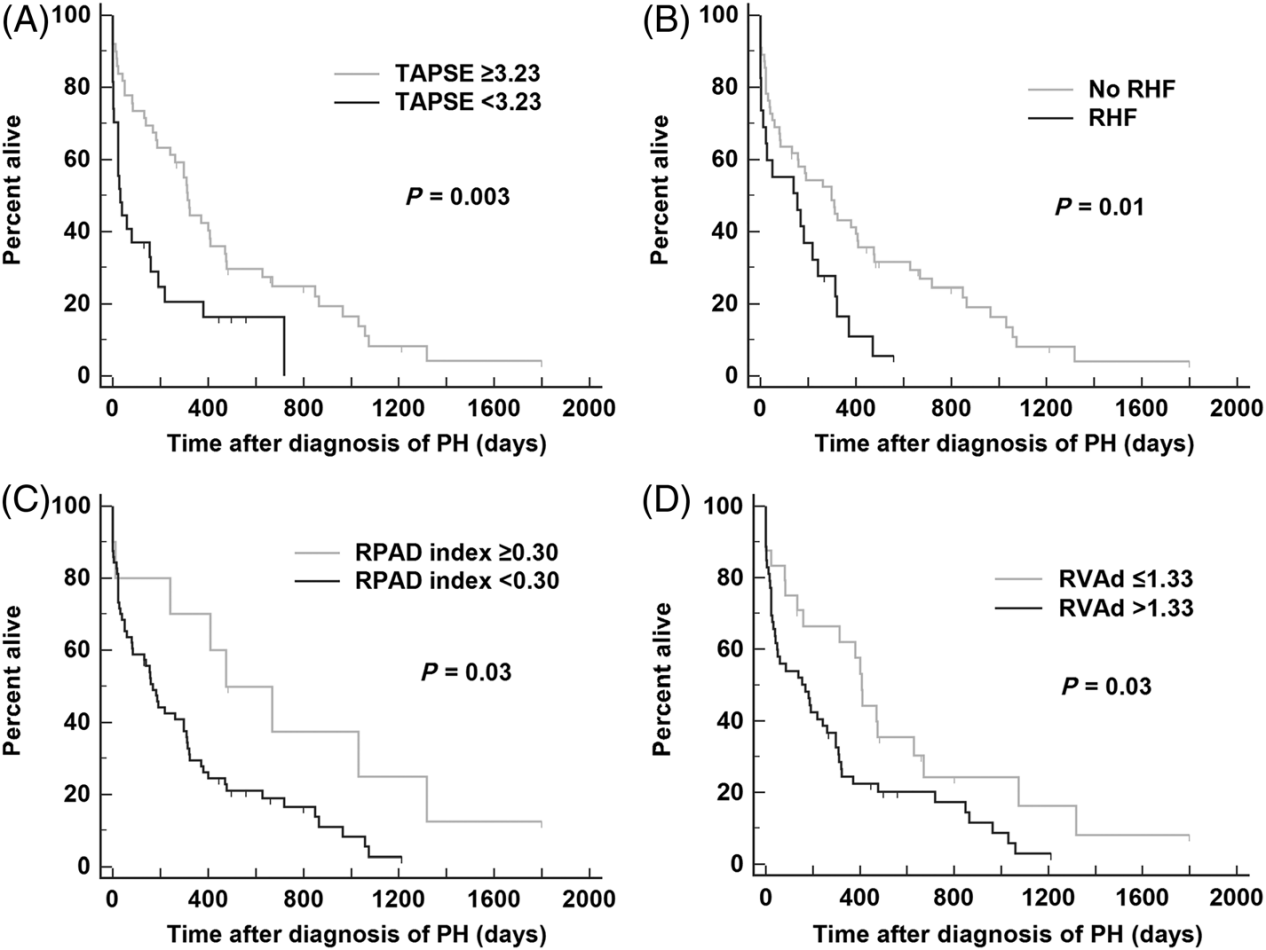
Kaplan‐Meier survival curves showing the significant effect (log‐rank test) of, A, TAPSE indexed to bodyweight in units of mm/kg^0.284^, B, RHF, C, RPAD index (unitless), and, D, RVAd indexed to bodyweight in units of cm^2^/kg^0.62^ on survival of dogs diagnosed with PH. PH, pulmonary hypertension; RHF, right heart failure; RPAD, right pulmonary artery distensibility; RVAd, right ventricular area at end‐diastole; TAPSE, tricuspid annular planes systolic excursion

**FIGURE 2:**
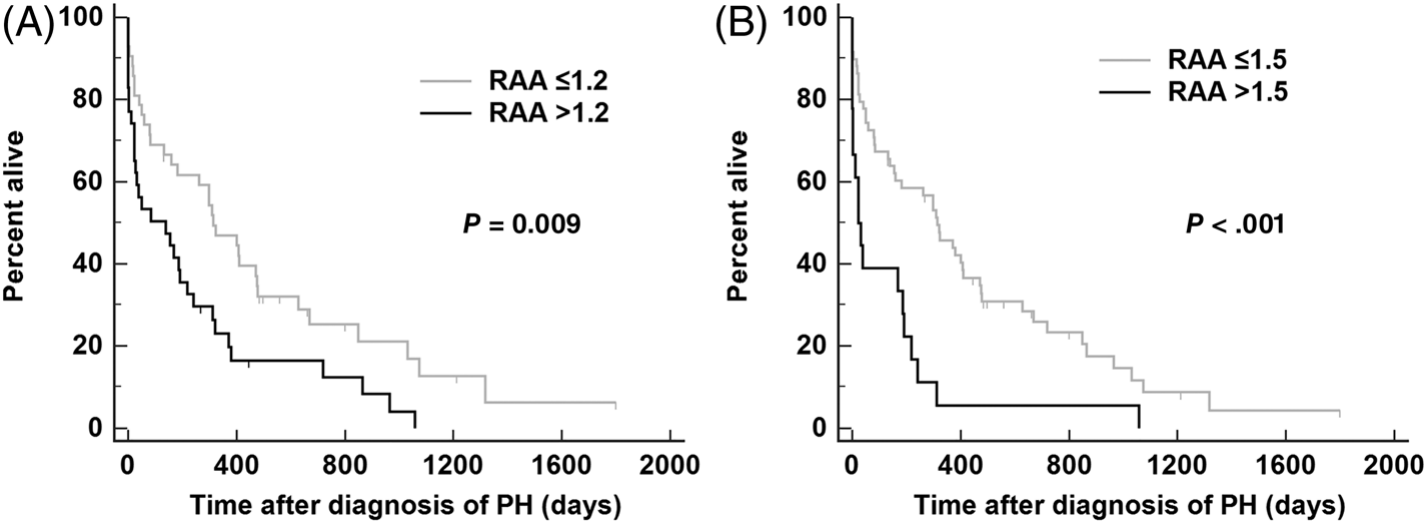
Kaplan‐Meier survival curves showing the significant effect (log‐rank test) of, A, RAA > 1.2 cm^2^/kg^0.71^ and, B, RAA > 1.5 cm^2^/kg^0.71^ on survival of dogs diagnosed with PH. PH, pulmonary hypertension; RAA, right atrial area

None of the echocardiographic variables were highly correlated based on Spearman's correlation analysis. The strongest correlation identified was between RAA and RVAd (*ρ* = 0.62, *P* < .001; the other *ρ* values ranged from 0.55 to 0.05). Of the 11 dichotomous variables evaluated as predictors of survival in the univariate analysis, right heart failure (*P* = .01), RVAd > 1.33 cm^2^/kg^0.62^ (*P* = .03), TAPSE < 3.23 mm/kg^0.284^ (*P* = .003), and RPAD index < 0.30 (*P* = .03) were significantly associated with worse outcomes (Figure 1 and Table 3). However, when these predictors (in addition to RAA > 0.76 cm^2^/kg^0.71^ and AT/ET < 0.30) were carried forward into the multivariable analysis, only right heart failure (*P* = .02) and TAPSE < 3.23 mm/kg^0.284^ (*P* = .04) had independent adverse associations with survival time (Table 3). When echocardiographic predictors were evaluated as continuous variables, TRV (*P* = .05), RAA (*P* = .007), RVAd (*P* = .02), RPAD index (*P* = .03), and AT/ET (*P* = .02) were significantly associated with worse outcomes (Table 4). When these predictors (in addition to TAPSE and right heart failure) were carried forward into the second multivariable analysis, only RAA (*P* = .02) was independently associated with survival time (Table 4). The post hoc analysis of RAA evaluated the effect of the 25th (0.9 cm^2^/kg^0.71^), 50th (1.2 cm^2^/kg^0.71^), and 75th (1.5 cm^2^/kg^0.71^) percentiles on survival. Results showed that the cutoffs of RAA > 1.2 cm^2^/kg^0.71^ (hazard ratio [HR] = 1.8 [95% CI 1.1‐2.9], *P* = .009) and RAA > 1.5 cm^2^/kg^0.71^ (HR = 3.6 [95% CI 1.8‐7.6], *P* < .001) were significantly associated with survival (Figure 2).

**TABLE 3:** Cox proportional‐hazards regression analyses to identify independent clinical or echocardiographic predictors of survival in 78 dogs^b^ with PH

Predictor variable	Univariate analysis		Multivariable analysis^a^
	HR (95% CI)	*P* value	*P* value
Age (y)	1.03 (0.95‐1.13)	.42	
PH secondary to LHD	0.93 (0.57‐1.53)	.78	
Right heart failure	2.05 (1.18‐3.57)	.01	.02
Syncope	1.09 (0.67‐1.77)	.73	
TRPG > 75 mm Hg	1.37 (0.84‐2.24)	.21	
RAA > 0.76 cm^2^/kg^0.71^	1.62 (0.76‐3.44)	.18	‐
RVAd > 1.33 cm^2^/kg^0.62^	1.82 (1.05‐3.15)	.03	‐
TAPSE < 3.23 mm/kg^0.284^	2.19 (1.28‐3.74)	.006	.04
PV/Ao > 1.0	1.42 (0.82‐2.47)	.20	
RPAD index < 0.30	2.31 (1.04‐5.12)	.02	.17
AT/ET < 0.30	1.61 (0.95‐2.74)	.07	‐

*Note:* This model utilized only dichotomous echocardiographic predictors. See Table 2 for the remainder of the key. “‐” = variables not included in the final model.

Abbreviations: CI, confidence interval; HR, hazard ratio.

^a^ Overall model *P* = .002.

^b^ 4 dogs were excluded from the survival analysis because they did not survive to discharge.

**TABLE 4:** Cox proportional‐hazards regression analyses to identify independent clinical or echocardiographic predictors of survival in 78 dogs^b^ with PH

Predictor variable	Univariate analysis		Multivariable analysis^a^
	HR (95% CI)	*P*‐value	*P*‐value
Age (y)	1.03 (0.95‐1.13)	.42	
PH secondary to LHD	0.93 (0.57‐1.53)	.78	
Right heart failure	2.05 (1.18‐3.57)	.01	.06
Syncope	1.09 (0.67‐1.77)	.73	
TRV (m/s)	1.60 (1.01‐2.53)	.05	‐
RAA (cm^2^/kg^0.71^)	2.72 (1.58‐4.70)	.007	.02
RVAd (cm^2^/kg^0.62^)	2.00 (1.14‐3.49)	.02	‐
TAPSE (mm/kg^0.284^)	0.85 (0.71‐1.02)	.06	‐
PV/Ao	0.91 (0.25‐3.26)	.88	
RPAD index	0.05 (0.003‐0.76)	.03	.06
AT/ET	0.02 (0.001‐0.55)	.02	‐

*Note:* This model utilized only continuous echocardiographic predictors. See Table 2 for the remainder of the key. “‐” = variables not included in the final model.

Abbreviations: Ao, aortic root; AT/ET, acceleration time to ejection time ratio of pulmonary artery flow; CI, confidence interval; HR, hazard ratio; LHD, left heart disease; PH, pulmonary hypertension; PV, pulmonary valve; RAA, right atrial area; RPAD, right pulmonary artery distensibility; RVAd, right ventricular area at end‐diastole; TAPSE, tricuspid annular plane systolic excursion; TRPG, tricuspid regurgitation pressure gradient.

^a^ Overall model *P* = .001.

^b^ 4 dogs were excluded from the survival analysis because they did not survive to discharge.

## 4 DISCUSSION

Our study showed that echocardiographic evidence of right heart enlargement is common in dogs with PH, regardless of the underlying etiology. Enlargement of the RA, RV, or PA was identified in at least 69% of all cases and RV systolic dysfunction was identified in one‐third of cases. Right ventricular function was significantly worse in dogs with PH not secondary to LHD when compared to dogs with PH secondary to LHD. However, survival times were not significantly different between dogs with PH secondary to LHD (ie, MMVD) and dogs with PH not secondary to LHD. Last, right atrial size (RAA) and decreased RV function (TAPSE < 3.23 mm/kg^0.284^) in addition to right heart failure were independently associated with reduced survival time in dogs with PH. These findings provide clinically relevant prognostic information and highlight the importance of quantifying right heart size and function (RAA and TAPSE, respectively) in dogs with PH related to any underlying cause.

Numerous studies have documented that RA enlargement (increased RAA),^19^ RV enlargement (increased RVAd),^20^ PA enlargement (increased PV/Ao),^7^, ^14^ and reduced RPAD index^14^, ^15^, ^16^, ^17^ are common in dogs with PH and that RV systolic dysfunction (reduced TAPSE) occurs in some dogs with PH.^21^, ^22^ Right ventricular enlargement and systolic dysfunction, PA enlargement or reduced RPAD index, and RA enlargement are considered to be clinically useful echocardiographic signs of PH and help assess the probability of PH.^2^ Our study confirms these findings and highlights the prognostic value of the echocardiographic measurements of RA size and RV systolic function.

Our study found that RV systolic dysfunction (TAPSE < 3.23 mm/kg^0.284^) was significantly less common in dogs with PH secondary to LHD/MMVD compared to PH due to other causes (PH not secondary to LHD). This represents an important finding that agrees with previous work evaluating TAPSE in dogs with PH and MMVD.^22^, ^35^, ^36^, ^37^ There appears to be general agreement that reduced TAPSE in dogs with PH secondary to MMVD is uncommonly detected or “masked,”^22^ as RV systolic dysfunction (quantified by TAPSE) appears be a poor predictor of PH in dogs with MMVD.^36^, ^37^ As previously hypothesized,^20^, ^36^ the influence of hyperdynamic LV systolic function in small breed dogs with MMVD,^38^ ventricular interdependence, and postcapillary PH (pulmonary venous hypertension) likely help explain the difference in RV function in dogs with PH secondary to LHD versus PH not secondary to LHD.

The current study represents an investigation evaluating the prognostic value of numerous echocardiographic measurements of right heart size and function in dogs with PH. A previous report^16^ evaluated the prognostic value of the RPAD index, right pulmonary artery size measurements, and TRPG in dogs diagnosed with PH and found only that TRPG > 38.9 mm Hg was independently associated with survival at 1 year. Our study revealed that the RPAD index (RPAD < 0.30 and RPAD as a continuous variable) and TRPG (TRV as a continuous variable) were significantly associated with survival based on univariate analyses. However, when these were carried forward into the multivariable analysis, statistical significance was lost. This variation between studies can likely be explained by differences in study population and study design (eg, enrollment criteria) and the additional clinical and echocardiographic variables evaluated in our study.

Our study emphasizes the importance of a comprehensive echocardiographic assessment of dogs with PH that goes beyond just measurement of TRV. The TRV/TRPG is undoubtedly a key component of the clinical assessment of dogs with suspected PH.^39^ However, our study suggests that additional quantitative measurements of right heart size and function might provide clinically useful prognostic information. Studies in humans with PH that also evaluated RAA^40^ and TAPSE^41^, ^42^ corroborate our findings. Estimates of pulmonary arterial pressures (TRV/TRPG) might be misleading when paired with severe RV systolic dysfunction.^43^ We suspect these indices (RAA and TAPSE) more accurately reflect the hemodynamic burden (afterload, that is, pulmonary vascular resistance) and RV‐arterial coupling (ie, matching of RV contractility to afterload) in dogs with PH when compared to estimates of pulmonary arterial pressure. Ventriculo‐arterial coupling appears to be an important metric in understanding RV adaptation to PH and thus functional status and prognosis.^44^ It accounts for both contractility and afterload. The RV is “coupled” when an increase in afterload is matched by an increase in RV contractility. Uncoupling occurs when increased afterload is not matched by increased RV contractility. This might explain why TRV and TRPG > 75 mm Hg failed to demonstrate an independent association with survival in our study. These metrics can only reflect RV afterload when RV contractility is normal or compensating. Echocardiographic metrics of RV systolic function (including TAPSE) have been shown to more closely represent ventriculo‐arterial coupling versus contractility (load‐independent).^45^

We used 2 different multivariable Cox proportional‐hazards regression models, 1 using previously established clinically relevant dichotomous echocardiographic predictor variables and 1 using continuous echocardiographic predictor variables. The latter suggested that RAA as a continuous predictor variable is significantly associated with risk of death and demonstrates that risk of death increases (at a constant increment) as RA size increases (at a constant increment). To attempt to categorize RA size in a more clinically relevant manner, we performed a post hoc analysis on RAA based on categorization into quartiles. It showed that dogs with RAA > 1.2 cm^2^/kg^0.71^ (50th percentile) are 2 times more likely to die than dogs with RAA ≤1.2 cm^2^/kg^0.71^; whereas dogs with RAA > 1.5 cm^2^/kg^0.71^ (75th percentile) are 3.6 times more likely to die than dogs with RAA ≤ 1.5 cm^2^/kg^0.71^. These cutoffs might be useful to help categorize RA enlargement (mild, moderate, severe) in a clinically relevant manner based on prognosis and relative risk of death in dogs with PH.

Our results are not applicable to all dogs with PH and should be interpreted within the context of the population studied. Specifically, dogs with a congenital shunting lesion and dogs with intracardiac heartworms were excluded. Dogs with a congenital shunting lesion were excluded because their younger age might have skewed survival data. Also, some shunting lesions can be corrected or cured, further skewing survival data. Dogs with intracardiac heartworms were also excluded because of the challenges of assessing or estimating increased PA pressure (ie, separating the effects of obstructive intracardiac heartworms versus increased PVR on estimated RV systolic pressure). Also, in our experience, outcome of these cases can range from being very poor (death during heartworm extraction) to excellent (with successful extractions). Thus, our study population largely consisted of dogs with acquired and likely chronic and irreversible PH where it is unlikely the underlying disease can be cured or corrected.

Given our study's retrospective design, all dogs did not have a consistent and thorough diagnostic evaluation nor could a definitive underlying cause of PH be determined to permit grouping of cases according to the cause of PH. Some causes of PH require invasive or impractical diagnostics for definitive diagnosis (eg, lung biopsy). Furthermore, classifying dogs into groups of PH is, in theory, intended to group dogs according to similar themes such as pathophysiology, treatment, and potentially prognosis.^2^ However, even when full diagnostic evaluations are performed, challenges are still encountered. Pathophysiology, treatment, and prognosis can vary considerably within a group. For example, group 1 PH (pulmonary arterial hypertension) consists of a diverse spectrum of diseases ranging from a left‐to‐right shunt, which can be cured (eg, a left‐to‐right shunting patent ductus arteriosus) to pulmonary veno‐occlusive disease or pulmonary capillary hemangiomatosis, which are largely diagnosed by necropsy and seem to have a very poor prognosis.^46^ Therefore, we elected to classify dogs with PH based on what was could be determined clinically, (ie, PH secondary to LHD versus PH not secondary to LHD) and avoid speculation on causes and grouping of PH based on variable and largely suboptimal diagnostic evaluations.

Results of our study should be interpreted within the context of its limitations. This was a retrospective study and therefore it lacked standardization of, for example, diagnostic evaluations and medications permitted. However, it should be noted that a minority of dogs enrolled in our study were taking medications known to affect the cardiovascular system (at most 32% were taking furosemide and only 4% were taking sildenafil). However, we recognize that this could have influenced our results to some degree. More importantly, as a retrospective study, treatment protocols for dogs after the diagnosis of secondary PH that might have impacted survival were not standardized. Our study had relatively strict enrollment criteria. We elected to follow recommendations from the ACVIM consensus guidelines and only enroll dogs with at least an intermediate probability of PH based solely on echocardiography,^2^ that is, dogs with a TRV > 3.4 m/s (TRPG > 46 mm Hg). This cutoff likely differs from previous studies on dogs diagnosed with PH, which are almost always less than 3.4 m/s. Also, it should be noted that only 1 dog enrolled in our study tested positive for heartworm and dogs with a congenital cardiac shunt were excluded. Therefore, these results are not broadly applicable to all dogs diagnosed with PH.

In conclusion, our study suggests that increased right atrial size (quantified by RAA), RV systolic dysfunction (based on two‐dimensional echocardiography‐derived TAPSE < 3.23 mm/kg^0.284^), and right heart failure are associated with an increased risk of death in dogs with PH. Quantitative indices of right heart size and function provide clinically relevant prognostic information and are important in dogs with PH.

## 5 CONFLICT OF INTEREST DECLARATION

Authors declare no conflict of interest.

## OFF‐LABEL ANTIMICROBIAL DECLARATION

Authors declare no off‐label use of antimicrobials.

## INSTITUTIONAL ANIMAL CARE AND USE COMMITTEE (IACUC) OR OTHER APPROVAL DECLARATION

Authors declare no IACUC or other approval was needed.

## HUMAN ETHICS APPROVAL DECLARATION

Authors declare human ethics approval was not needed for this study.
